# Time-Aware Service Ranking Prediction in the Internet of Things Environment

**DOI:** 10.3390/s17050974

**Published:** 2017-04-27

**Authors:** Yuze Huang, Jiwei Huang, Bo Cheng, Shuqing He, Junliang Chen

**Affiliations:** State Key Laboratory of Networking and Switching Technology, Beijing University of Posts and Telecommunications, Beijing 100876, China; huangyz@bupt.edu.cn (Y.H.); chengbo@bupt.edu.cn (B.C.); hsq@bupt.edu.cn (S.H.); chjl@bupt.edu.cn (J.C.)

**Keywords:** time series analysis, quality of service (QoS), service ranking prediction, Internet of things (IoT)

## Abstract

With the rapid development of the Internet of things (IoT), building IoT systems with high quality of service (QoS) has become an urgent requirement in both academia and industry. During the procedures of building IoT systems, QoS-aware service selection is an important concern, which requires the ranking of a set of functionally similar services according to their QoS values. In reality, however, it is quite expensive and even impractical to evaluate all geographically-dispersed IoT services at a single client to obtain such a ranking. Nevertheless, distributed measurement and ranking aggregation have to deal with the high dynamics of QoS values and the inconsistency of partial rankings. To address these challenges, we propose a time-aware service ranking prediction approach named TSRPred for obtaining the global ranking from the collection of partial rankings. Specifically, a pairwise comparison model is constructed to describe the relationships between different services, where the partial rankings are obtained by time series forecasting on QoS values. The comparisons of IoT services are formulated by random walks, and thus, the global ranking can be obtained by sorting the steady-state probabilities of the underlying Markov chain. Finally, the efficacy of TSRPred is validated by simulation experiments based on large-scale real-world datasets.

## 1. Introduction

The Internet of things (IoT) is an infrastructure that interconnects uniquely identifiable sensors through the Internet [1]. IoT systems consist of numerous of IoT applications (also called IoT services), each of which acquires the data collected from the geographically dispersed sensors, and is composed of a series of atomic services providing simple functionalities [2]. Thus, during the procedures of building the IoT systems, users should search the atomic services, and compose them to accomplish a certain goal or constitute new applications [3]. With the rising popularity of IoT, how to build high-quality IoT systems is an urgent requirement in both academia and industry.

In the process of building high-quality IoT systems, once the number of functional similar services increases dramatically, optimal service selection according to the nonfunctional performance becomes important. Nonfunctional performance is usually described by quality of service (QoS), which consists of many attributes, including response time, throughput, reliability, availability, security, etc. QoS can provide valuable information for services selection and recommendation. QoS service attributes will be affected by many factors, i.e., large-volume data will be processed by the applications [4] and the transmission delay of the services etc., therefore, the QoS presents the temporal dynamic characteristic accordingly. Thus, the study of QoS temporal dynamic changes is the most important issue in the IoT environment.

For building IoT systems, atomic IoT services should be selected to compose the applications with complex functionality. Once a set of IoT services that fulfills the requested functionality is discovered, the order of the candidate services needs to be calculated according to the QoS values [5]. Service ranking is the procedure of rating the candidate services, and providing the order of the services according to a certain rule. The most straightforward approach of service ranking is by rating all the candidate services at the user side and ranking the services according to the numerical QoS values. With the increasing of services, it is difficult to evaluate all the services at the client side, as it is a time-consuming and resource-consuming process for invoking all the services, therefore, the service ranking prediction is presented to reduce the cost of ranking the services [6].

Due to the different environment of the clients or different adopted rating criteria, the rating of the candidate services may not be consistent from one client to another. Therefore, how to obtain the global ranking of all the services from the clients is a challenging issue. In our previous research, we proposed an approach for obtaining the global ranking by pairwise comparison model [7]. This method not only can obtain the global ranking from collection of partial rankings, but also can lower the storage space of training data in comparison with that of the work [6]. It mainly focused on how to obtain the global service ranking, ignoring the differentials of the QoS value and the temporal dynamic changes of QoS. Therefore, how to obtain the global service ranking by studying the temporal dynamic changes of QoS is an important and unexplored problem for IoT service ranking.

To fill this gap, we present a time-aware service ranking prediction approach named TSRPred. We use a pairwise comparison model to describe the relationships between different services, and forecast future QoS comparison values by time series analysis method, and then the comparisons of IoT services are modeled by random walks. Furthermore, the global ranking is obtained by sorting the steady-state probabilities of Markov chain. Finally, the effectiveness of our approach is validated by the simulation experiments based on large-scale datasets. More specifically, the contributions of this paper are three-fold as follows:

(1) The time-aware service ranking prediction approach is proposed to obtain the global ranking, which can obtain the service ranking by studying the temporal dynamic changes of QoS.
(2) During the process of our approach, the temporal dynamic changes of QoS attributes are studied by time series forecasting method, which can forecast the future values and dynamic trends using fitted models.
(3) A random walk model is constructed based on pairwise comparison model, which is used to obtain the global service ranking from collection of partial rankings by considering the differentials of QoS values.

The remainder of this paper is organized as follows. Section 2 presents the basic concepts and definitions used in this paper. Section 3 proposes the overall framework of our service ranking prediction method, and then the model of service ranking is introduced in this section. Section 4 presents the method and algorithm for global ranking. Section 5 introduces a case study to investigate the benefit of our approach. Furthermore, we evaluate the effectiveness of our approach in Section 6. Finally, we introduce the related work of this research in Section 7 and conclude this paper in Section 8.

## 2. Preliminaries

In this section, we introduce concepts and definitions used in this paper. Firstly, we formulate the basic form of QoS, which is the input of our approach, and then the basic definition of time series model is formulated.

**Figure 1:**
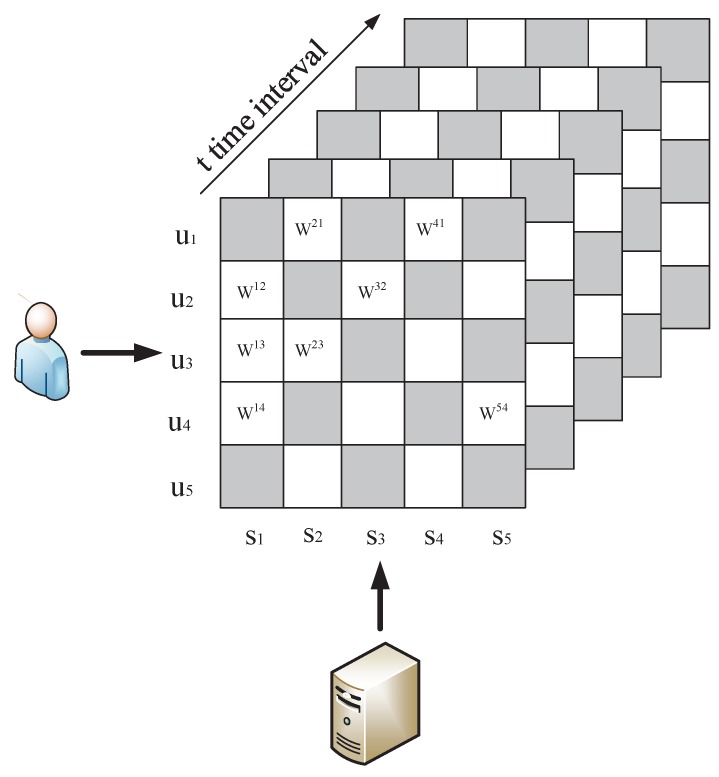
Quality of service (QoS) Matrix.

In this paper, we need analyze the QoS dataset to obtain the global service ranking. Assume there are *n* IoT services $S=\{s_{1},s_{2},...,s_{n}\}$ invoked by *m* users $U=\{u_{1},u_{2},...,u_{m}\}$. Each service has its QoS attributes monitored over some time, which include response time, throughput, etc. When a user invokes a IoT service, we can obtain the QoS information during *t* time intervals. By integrating all the QoS information from users, we form a three-dimensional user-service-time matrix as shown in Figure 1. Thus each entry denotes a series-observed QoS value $W=\{w_{1}^{ij},w_{2}^{ij},...,w_{t}^{ij}\}$ of an IoT service $s_{i}$ invoked by a user $u_{j}$ over the specific time intervals.

As aforesaid, we study the temporal dynamic changes of QoS attributes through analyzing the QoS dataset. In this paper, we adopt time series prediction models to analyze the QoS matrix, which were used in different sectors such as stock trend prediction, meteorological data analysis, etc. During this procedure, the most important step was construction of a time series model to fit the original data. From wide review of literature studied the time series models, there exist different time series prediction models that work on different patterns, e.g., the autoregressive (AR) model, moving average (MA) model, and autoregressive integrated moving average (ARIMA) model, etc. The ARIMA model is the most widely used model for time series forecasting, which was proposed by Box and Jenkins [8], and can be formulated as follows.

As Definition 1 shows, the most important problem for constructing the fitted time series model is how to determine the order of *p*, *q*, and *d*. During this procedure, if the original time series $\left\{x_{t}|t=1,2,...,n\right\}$ is non-stationary, *d* differences should be done to transform the data into a stationary series. Consequently, $\left\{x_{t}|t=1,2,...,n\right\}$ is said to be an ARIMA model denoted by ARIMA(*p*, *d*, *q*). Therefore, we need to determine the order of *p*, *d*, and *q* during the process of time series forecasting, which will be discussed in Section 3.

**Definition** **1** (ARIMA Model)**.** *The autoregressive integrated moving average (ARIMA) model is a widely discussed model in time series forecasting, which integrates both the autoregressive (AR) model and moving average (MA) model. A non-stationary time series $\left\{x_{t}|t=0,1,2,...,n\right\}$ can be transformed into the stationary time series after d differences; the series can be modeled by the autoregressive average (ARMA) model, denoted by ARMA(p,q), if it satisfies:*

(1) $$x_{t}=\phi_{0}+\sum_{i=1}^{p}\phi_{i}x_{t-i}+\sum_{j=1}^{q}\theta_{j}a_{t-j}+a_{t}$$

*where, $\phi_{0}$ is the constant term, and $\phi_{i}$ and $\theta_{j}$ are the parameters for the AR model and MA model, respectively. $a_{t}$ is the random error, which is a white noise. The non-negative integer p, q and d denote the order of AR, the order of MA, and the order of difference, respectively.*

## 3. Model of Service Ranking Prediction

### 3.1. Framework

**Figure 2:**
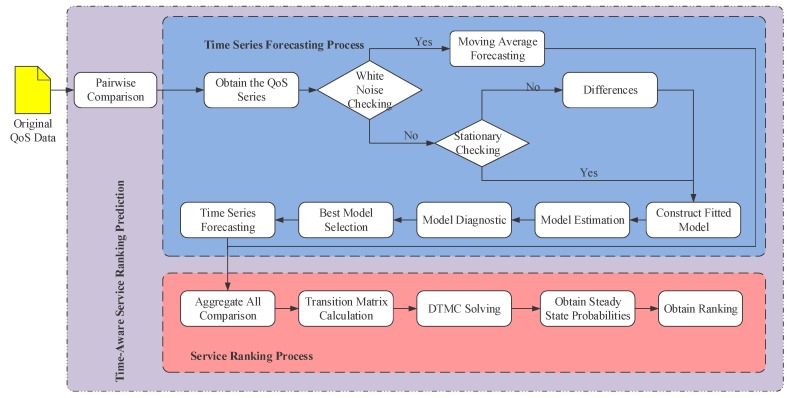
Framework of service ranking prediction. QoS: quality of service; DTMC: discrete-time Markov chain.

In this context, we put forward an approach to predict the global service ranking from partial service rankings, namely TSRPred, which can be decomposed into the following three phases, including pairwise comparison, time series forecasting, and service ranking. The framework of TSRPred is illuminated by Figure 2.

As Figure 2 shows, during the procedure of our approach, we obtain the original QoS data collected from the candidate IoT services at first, and then we compare the services by pairwise comparison model, which can fill the gap of inconsistent measurements. Once the pairwise comparison model is constructed, the comparisons are transformed to the QoS future value forecasting. Therefore, the partial rankings can be obtained by time series forecasting method. Furthermore, a random walk approach is adopted as means to reach the rank aggregation from the collection of all partial rankings. Finally, the steady-state probabilities of the discrete-time Markov chain is calculated and sorted to obtain the global service ranking. In the following context, each phase of our approach will be introduced in detail.

### 3.2. Pairwise Comparison Model

In our service ranking prediction method, the main idea is to collect the partial rankings and obtain the global service ranking. It is acknowledge that invoking all the services from a single client is time- and resource-consuming, therefore it is difficult to rate all the candidate services efficiently from a single client; another non-ignorable factor is the inconsistent of different rating measurements. To address these problems, an efficient approach is to obtain the global service ranking by aggregating all partial service rankings. In this paper, we propose a pairwise comparison model to get the partial rankings [9], which cannot rely on any service ranking scheme.

**Figure 3:**
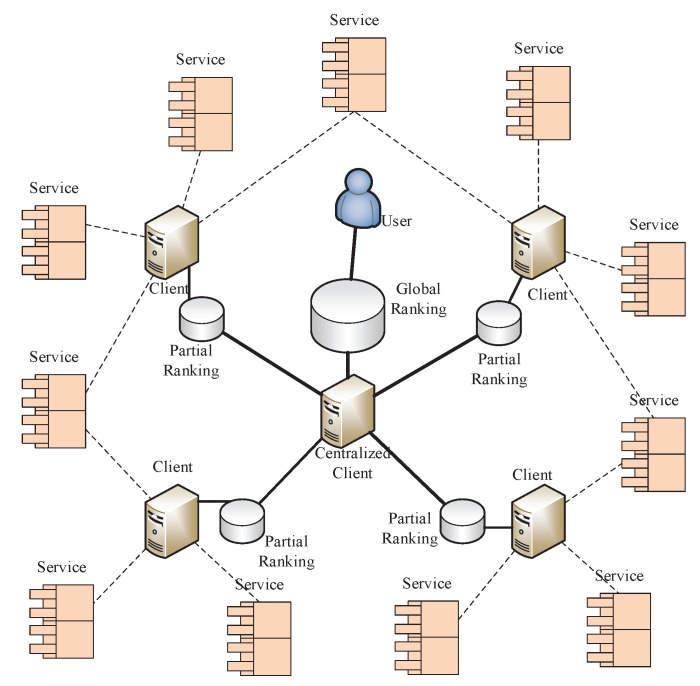
Pairwise comparison model.

In this model, we focus on how to collect all partial rankings to obtain the global service ranking. The model demonstration is shown in Figure 3. It is assumed the set of candidate IoT service contains *n* IoT services with similar functionality, which are geographically dispersed in different locations. In sight of this characteristic, we deploy *m* different clients to rate these services, and each of them only rates a portion of the candidate services. Thus the partial ranking of each subset service is rated at a client, finally, a centralized client is used to collect all the partial rankings and obtain the global service ranking. We note that, in such a model, the detail ranking criteria can be ignored, and the user should only focus on the process for aggregating partial rankings.

The pairwise comparison model reflects the relationship between different services, which have been discussed by previous work [10]. In this paper, it is assumed two candidate IoT services need to be ranked, which are $s_{i}$ and $s_{j}$, and there is a weight score $w_{i}\in R^{+}$ associated with service $s_{i}$, which can be regarded as the QoS attributes, i.e., response time, throughput, etc. Therefore, the *l*-th comparison outcome $P_{ij}^{\left(l\right)}$ of the ranked IoT services $s_{i}$ and $s_{j}$ can be expressed as the following form:

(2) $$\begin{array}{c} P_{ij}^{\left(l\right)}=\left\{\begin{array}{cc} 1, & \mathrm{with}\mathrm{probability}\;\frac{w_{j}}{w_{i}+w_{j}};\\ 0, & \mathrm{with}\mathrm{probability}\;\frac{w_{i}}{w_{i}+w_{j}}. \end{array}\right. \end{array}$$

As Equation (2) shows, if $P_{ij}^{\left(l\right)}=1$ represents the service, $s_{j}$ is ranked higher than service $s_{i}$. We notice that, in our setting, $P_{ij}^{\left(l\right)}$ are independent of each other for the services $s_{i}$, $s_{j}$, and *l*.

As discussed in our previous work [7], the relationship between two IoT services can be denoted as a directed graph G=(V,E), where *V* represents the set of candidate services used to be ranked, and *E* represents the edges of these services. Here, the transmission from $s_{i}$ to $s_{j}$ can be denoted as $\left(s_{i},s_{j},q_{ij}\right)$, where the $q_{ij}$ indicates the probability service $s_{j}$ is ranked higher than $s_{i}$. We can obtain the value of $q_{ij}$ by the following equation:

(3) $$q_{ij}=\frac{1}{k}\sum_{l=1}^{k}P_{ij}^{\left(l\right)}$$

where *k* is the number of comparisons between service $s_{i}$ and service $s_{j}$. In Equation (3), we let $q_{ij}=q_{ji}=0$, when the pair of $s_{i}$ and $s_{j}$ has not been compared.

Compared with the previous work [7], our model focuses on the differentials of QoS, and considers the differentials of QoS for candidate services $s_{i}$ and $s_{j}$. Note that by the strong law of large numbers (SLLN), for independent and identically distributed (i.i.d.) random $P_{ij}^{l}$’s, when k→∞, the $q_{ij}$ converges to the corresponding weights of the compared services.

(4) $$\begin{array}{cc} \lim_{k\to \infty }q_{ij}=E\left[P_{ij}^{\left(l\right)}\right] & =\frac{w_{j}}{w_{i}+w_{j}}\\ & =\frac{\frac{1}{2}w_{i}+\frac{1}{2}w_{j}+\frac{1}{2}\left(w_{j}-w_{i}\right)}{w_{i}+w_{j}}\\ & =\frac{w_{i}+w_{j}+w_{j}-w_{i}}{2(w_{i}+w_{j})}\\ & =\frac{1}{2}+\frac{w_{j}-w_{i}}{2(w_{i}+w_{j})} \end{array}$$

Therefore, the $q_{ij}$ can be expressed as the following form:

(5) $$\begin{array}{c} q_{ij}=\left\{\begin{array}{cc} \frac{1}{2}+\frac{w_{j}-w_{i}}{2(w_{i}+w_{j})}, & \mathrm{if}\;i\neq j;\\ 0, & \mathrm{if}\;i=j; \end{array}\right. \end{array}$$

where *k* is the number of comparisons between service $s_{i}$ and service $s_{j}$ for all clients.

As discussed in our previous work [7], the comparisons between different services can be represented as random walks on the directed graph *G*; in such a graph, we use $p_{ij}$ to indicate the transition probability from service $s_{i}$ to $s_{j}$. tTe $p_{ij}$ can be denoted as the following form:

(6) $$\begin{array}{c} p_{ij}=\left\{\begin{array}{cc} \frac{1}{d_{\max}}q_{ij}=\frac{1}{d_{\max}}\left[\frac{1}{2}+\frac{w_{j}-w_{i}}{2(w_{i}+w_{j})}\right], & \mathrm{if}\;i\neq j;\\ 1-\frac{1}{d_{\max}}\sum_{m\neq i}q_{im}=1-\frac{1}{d_{\max}}\sum_{m\neq i}\left[\frac{1}{2}+\frac{w_{m}-w_{i}}{2(w_{i}+w_{m})}\right], & \mathrm{if}\;i=j; \end{array}\right. \end{array}$$

where $d_{max}$ is the maximum out-degree of a node. In order to ensure the random walk process is stable by satisfying $\sum_{j}p_{ij}=1$, we add the self-loops in this graph.

Since the transition probability of each state is obtained, the goal of service ranking prediction is transform to QoS comparison value forecasting. Next, we will discuss the time series forecasting in detail.

### 3.3. Time Series Forecasting

In order to obtain the global service ranking, we need forecast the QoS future values. To our best knowledge, the QoS attributes present the typical temporal dynamic characteristic in the IoT environment. Time series forecasting is a widely adopted method to study the temporal dynamic characteristics by analyzing the history data.

During the procedure of time series forecasting, the most important step is how to determine the fitted time series model for modeling and forecasting the data. In this paper, we adopt the ARIMA model for QoS differentials forecasting. The process of time series forecasting can be decomposed into following steps [8].

Step 1 White noise checking. Before constructing ARIMA models, we should check whether the original time series data has white noise. If they do not satisfy the condition of white noise, we need perform the following steps, otherwise, the simple moving average approach is adopted to obtain the future values.
Step 2 Stationarity checking. The stationarity checking is the pre-condition of model identification. If the time series has non-stationarity, *d* differences should be done to transform the original time series into a stationary series.
Step 3 Model identification. In this step, the key issue is how to determine the order of *p* and *q*. We need determine the concrete orders of the ARIMA model according to the observation of autocorrelation function (ACF) and partial autocorrelation function (PACF).
Step 4 Model estimation. After we determine the order for ARIMA, we need determine the parameters of identified models to provide the best fit to the time series data.
Step 5 Model checking. Model checking involves the diagnostic checking for model adequacy. In this process, we should check the significance of the candidate models and their associated parameters.
Step 6 Model selection. Once all candidate models are estimated and checked, the best model is selected based on Akaike’s information criterion (AIC).
Step 7 Forecasting. Since the ARIMA model is modeled, the future QoS differentials can be obtained according to the fitted model.

*Step 1: White Noise Checking:* The time series forecasting method is constructed based on the assumptions of the time series, which are serial dependency, normality, stationarity, and invertibility [11]. In order to ensure the time series data can be effectively characterized by time series models, it must satisfy the serial dependency over the observed time period. If the time series data does not show serial dependency (also called white noise), the time series models cannot be used to model and forecast the future data. The widely used approach for white noise checking is the Ljung-Box test [12]. In the Ljung-Box test, the LB-statistics can be regarded as conforming the $\chi^{2}$ distribution approximately, which can be denoted as the following form:

(7) $$LB=n\left(n+2\right)\sum_{k=1}^{m}\left(\frac{{\hat{\rho_{k}}}^{2}}{n-k}\right)\dot{∼}\chi^{2}\left(m\right)$$

where *n* is the number of observed step, and *m* represents the number of lag. When the LB-statistics are larger than the quantile of $\chi_{1-\alpha }^{2}\left(m\right)$ or the p-value is smaller than α, the series can be regarded as a serial dependency time series, otherwise the series is white noisy.

In our approach, if the series is white noisy, we adopt the simple moving average approach to obtain the future values. In a simple moving average approach, the average of historical observations can be regarded as the next step forecasting value, which can be expressed as follows:

(8a) $$\begin{array}{cc} {\hat{x}}_{n+1} & =\frac{x_{n}+...+x_{1}}{n} \end{array}$$

(8b) $$\begin{array}{cc} {\hat{x}}_{n+2} & =\frac{{\hat{x}}_{n+1}+x_{n+1}+x_{n}+...+x_{1}}{n+1}\\ •\\ •\\ • \end{array}$$

(8c) $$\begin{array}{cc} {\hat{x}}_{n+l} & =\frac{{\hat{x}}_{n+l-1}+{\hat{x}}_{n+l-2}+...+{\hat{x}}_{n+1}+x_{n}+...+x_{1}}{n+l-1} \end{array}$$

where ${\hat{x}}_{n+l}$ represents the *l*-th forecasting value of the series $\left\{x_{t}|t=1,2,...,n\right\}$, and $x_{n}$ represents the *n*-th historical observation of $\left\{x_{t}|t=1,2,...,n\right\}$.

*Step 2: Stationarity Checking:* Since the white noise checking for QoS time series is completed, the stationarity checking should be done to determine the stationarity of the time series. The unit root test is the widely used approach for stationarity checking, which includes the DF test, ADF test, PP test, KPSS test, ERS test and NP test [13]. In this paper, we employ the ADF test to check the stationarity of QoS series, where the ADF-statistics denoted as τ can be expressed as (9):

(9) $$\tau =\frac{\hat{\rho }}{S(\hat{\rho })}$$

where $S(\hat{\rho })$ represents the sample standard error of parameter ρ. When the ADF statistics are larger than the critical value, the QoS series is non-stationary. Therefore, *d* differences should be done to transform the time series into the stationary series.

*Step 3: Model Identification:* As Definition 1 shows, after the white noise checking and stationary checking of QoS series is finished, we should identify the time series model. In this step, the most suitable orders of *p* and *q* for ARIMA model should be selected. The observation of autocorrelation function (ACF) and partial autocorrelation function (PACF) of the time series can help to make this selection.

Generally speaking, the lag *k* autocorrelation function (ACF) denoted as $\rho_{k}$ is defined by:

(10) $$\rho_{k}=\frac{\gamma_{k}}{\sigma^{2}}$$

where $\gamma_{k}=E\left[\left(x_{t}-\mu \right)\left(x_{t+k}-\mu \right)\right]$ represents the lag *k* autocovariance function.

The definition of the partial autocorrelation function (PACF) follows naturally from Equation (10). The lag *k* partial autocorrelation function denoted as $\phi_{kk}$ is defined by:

(11) $$\phi_{kk}=\frac{\rho_{k}-\sum_{j=1}^{k-1}\phi_{k-1,j}\rho_{k-j}}{1-\sum_{j=1}^{k-1}\phi_{k-1,j}\rho_{j}}$$

where $\phi_{k,j}=\phi_{k-1,j}-\phi_{kk}\phi_{k-1,k-j}$ and j=1,2,...,k−1.

When we make the selection of the orders of ARIMA models, the observation of ACF and PACF can help us to select the concrete orders of *p* and *q*. The selection according to the ACF and PACF characteristics can be found in Table 1.

In Table 1, if the ACF presents decay and PACF presents p-order cutting off, the AR(*p*) model is selected to model the QoS series. If ACF presents q-order cutting off while PACF presents decay over time, the MA(*q*) is selected to be the fitted model. If ACF and PACF curve decays, the ARMA(*p*,*q*) is selected.

*Step 4: Model Estimation:* Since the ARIMA models are selected, the parameters of identified models should be estimated in this step. Maximum likelihood estimation is the widely used approach for parameter estimation [8]. The likelihood function denoted as *l* can be expressed as following.

(12) $$l\propto {\left(\sigma^{2}\right)}^{-\frac{n}{2}}exp\left\{-\frac{1}{2\sigma^{2}}\sum_{t=1}^{n}{\left(a_{t}\right)}^{2}\right\}$$

where $a_{t}∼N\left(o,\sigma^{2}\right)$ is the white noise in the ARIMA model. In Definition 1, the value of parameters $\phi_{i}$ and $\theta_{j}$ that maximize the likelihood function *l* are referred to during the process of maximum likelihood estimation.

*Step 5: Model Checking:* During the process of model checking, we involve diagnostic checking for model adequacy, which includes the model significance test and parameters significance test. If one diagnostic is not satisfied, the current model is inadequate and should be removed from the candidate models.

For the model significance test, we adopt the Box-Ljung test to check whether the model satisfies the requirement. As Equation (7) shows, if the LB-statistics are smaller than the quantile of $\chi_{1-\alpha }^{2}\left(m\right)$ or the *p*-value is larger than α, the fitted model regarded as most significant can be selected. For the parameter significance test, we adopt the t-statistics to check whether the parameters are significantly non-zero. The t-statistics denoted by *T* can be expressed as following:

(13) $$T=\sqrt{n-m}\frac{\hat{\beta_{j}}}{\sqrt{a_{jj}Q\left(\tilde{\beta }\right)}}∼t\left(n-m\right)$$

where $\hat{\beta_{j}}∼N\left(0,a_{jj}\sigma_{\varepsilon }^{2}\right)$ is the least square estimation of *j*-th unknown parameter $\tilde{\beta }$, and $Q(\tilde{\beta })$ is the minimum sum of squared residuals of $\tilde{\beta }$. If the p-value is smaller than α, we ought to reject the null hypothesis that the parameter is significant.

*Step 6: Model Selection:* Once all candidate models are estimated and checked, the best model is selected based on Akaike’s information criterion (AIC) [14], which is denoted by:

(16) AIC=2k−2ln(l)

where k=p+q+1 represents the number of parameters, and *l* is the maximized value of the likelihood function of the estimated model. The process of model selection uses the minimum AIC value, so the model with the minimum AIC value is the best model, which will be selected to model and forecast the future QoS differentials.

*Step 7: Forecasting:* When the ARIMA model is constructed and all of the parameters are estimated, the future QoS differentials can be obtained according to the fitted model. Note that the *n*-th forecasting value is estimated based on the (n−1)-th forecasting value, so with the increase of forecasting steps, error increases.

In summary, the future QoS differentials are forecasted by the time series model, which indicates the partial rankings between different services. In order to obtain the global service ranking, we need to obtain the global ranking for collecting partial rankings. Here we construct the Markov model for obtaining the global rankings, which will be explained in Section 3.4.

### 3.4. Markov Model for Random Walks

As described in Section 3.2, the probability of each transition in random walk model represents the weight of the corresponding service, and the ranking between the the steady-state probabilities indicates the global service ranking, thus the global service ranking can transform to the steady-state probabilities ranking. In this section, we propose a Markov model-based approach to derive the global ranking.

**Figure 4:**
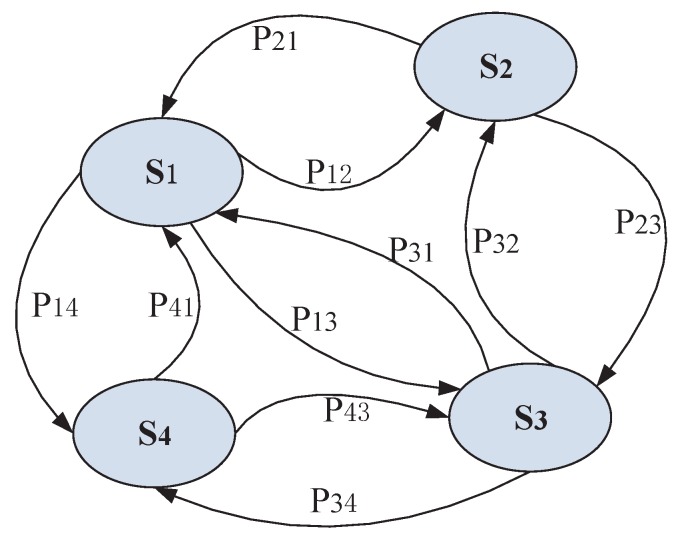
Discrete-time Markov chain demonstration.

With the random walk model presented above, in order to obtain the global ranking from the collection of partial rankings, the random walk can be regarded as a discrete-time Markov chain (DTMC). In the Markov model the transition matrix *P* is time-independent, where the transition probability $Pr_{ij}$ can be expressed as the following form, and the demonstration of DTMC can be seen in Figure 4.

(15) $$Pr_{ij}=Pr\left(S_{t+1}=j|S_{t}=i\right)=p_{ij}$$

where $p_{ij}$ represents the transition probability which is obtained from Equation (6).

In this paper, we use $\pi =[\pi_{1},\pi_{2},...,\pi_{n}]$ represent the steady-state probabilities in DTMC, which have the relationship as $\sum_{i=1}^{n}\pi_{i}=1$. Therefore, the steady-state probabilities can be obtained from the following formula:

(16) π·P=π

## 4. Algorithms for Obtaining Global Ranking

In the previous section, a pairwise comparison model is constructed to obtain the partial service rankings, where the QoS differentials are forecasted by time series models. Furthermore, the discrete-time Markov chain based on random walks is modeled to derive the global ranking. In this section, we will detail the algorithms for obtaining global ranking.

Our approach for obtaining service ranking can shield the methodologies of how to rate the services, and obtain the global service ranking with limits and noise information. The procedures for obtaining global ranking are illuminated as follows.

Step 1 In the first step, our approach selects all services pairs based on the constructed pairwise comparison model.
Step 2 The future values of QoS differentials can be estimated by the fitted time series model for obtaining the partial service rankings.
Step 3 All partial rankings are aggregated and the transition matrix is calculated by the formula (6).
Step 4 Furthermore, DTMC with transition matrix *P* can be solved by π·P=π.
Step 5 Finally, the global service ranking is derived through steady-state probabilities ranking.

In order to obtain the partial rankings, we construct pairwise comparison, where the future QoS differentials are estimated by the time series model. The detailed procedures are illustrated in Algorithm 1. As discussed in Section 3.3, after performing the white noisy checking and stationary checking for QoS differentials, we need construct the time series models to fit the QoS series, and estimate the associated parameters of the fitted time series models. Since the models are fitted and parameters are estimated, all of them must be checked by significance tests. Furthermore, the best model is selected based on Akaike’s information criterion (AIC). Finally, the future QoS differentials are estimated by fitted time series model, and thus the partial rankings are obtained accordingly.

**Algorithm 1** Algorithm for time series forecasting**Input:** QoS data *D* **Output:** Predicted QoS values $\left\{q_{t}^{ij}|t=n,n+1,...,n+m\right\}$
1:Analyze the QoS data *D*. Services pairs are selected out based on the pairwise comparison model, the comparison is obtained for each pair $\left\{\left(s_{i},s_{j}\right)|q_{t}^{ij}=\frac{1}{2}+\frac{w_{i}-w_{j}}{2(w_{i}+w_{j})},t=1,2,...,n\right\}$2:**for** Each service pairs **do**3: **if** the p-value of LB-test $pv_{lb}<\alpha$ **then**4:  This series has serial dependency5:  **if** the p-value of the ADF test $pv_{adf}<\alpha$ **then**6:   $\left\{q_{t}^{ij}\right\}\leftarrow \mathrm{diff}\left(\left\{q_{t}^{ij}\right\}\right)$7:  **end if**8:  Identify the models for QoS series9:  Estimate the parameters of the identified models10:  Check the significance of all candidate models, remove the non-significance models from the candidate models11:  Select the best model from significance models as the fitted model12:  Obtain the predicted QoS comparison values $\left\{q_{t}^{ij}|t=n,n+1,...,n+m\right\}$ by time series forecasting13: **else**14:  Forecast the future QoS comparison values by (8)15: **end if**16:**end for**


During the procedures for obtaining global service ranking, the most important step is to solve the DTMC with transition matrix *P* and obtain the steady-state probabilities π. In prior research, some efficient approaches, such as multiple relatively robust representations (MRRRs) are proposed for solving DTMC [15]. Here we adopt an iterative algorithm to obtain the steady-state probabilities. In our algorithm, an arbitrary $\pi_{0}$ subjected $\left|\right|\pi_{0}{\left|\right|}_{1}=1$ is selected at first, and then the $L_{2}$-norm is applied to find the probabilities iteratively. Detailed information can be found in our previous work [7].

## 5. Case Study

In this section, a case study of time series forecasting is introduced at first, and then the Markov chain is constructed based on the forecasting values. Furthermore, a prototype system framework and case study in reality are presented to investigate the benefit of our approach.

### 5.1. Example of Service Ranking

In this paper, the QoS temporal dynamic characteristics are studied by time series forecasting, and the global service ranking is derived through steady-state probabilities ranking. Here, we will give an example to show the basic procedures of our approach.

We use a benchmark QoS dataset [16,17] of 4500 real-word services, which are invoked by 142 users over 16 h with a time interval lasting for 15 min. Here we select the response time in this dataset. We assume there are two clients and each of them invokes three services. Services with ID 4386, 4009 and 3242 are invoked by client 1, while services with ID 1, 4386 and 4009 are invoked by client 2.

**Figure 5:**
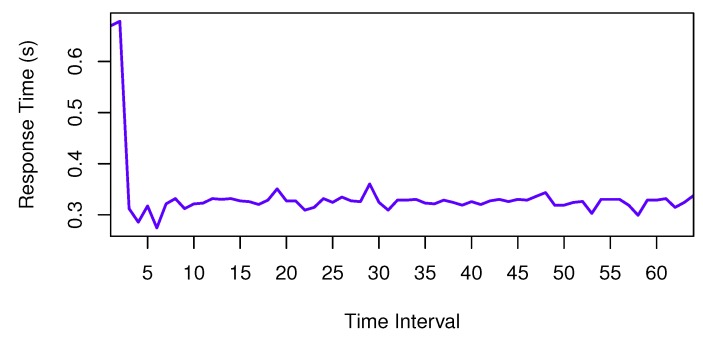
QoS Series.

At first, we use the comparison between services with ID 4386 and 4009 invoked by client 1 to investigate the procedure of time series forecasting. In our approach, we should analyze the QoS comparison of ranked services. The QoS comparison series is shown in Figure 5.

**Figure 6:**
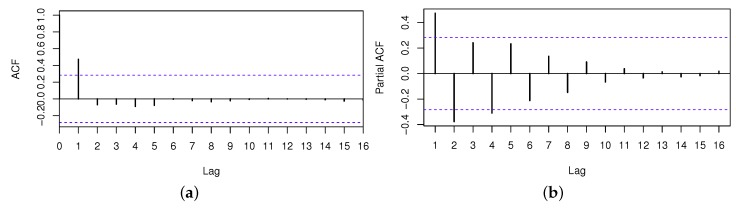
ACF and PACF of the QoS series. (**a**) ACF of the QoS series; (**b**) PACF of the QoS series.

As discussed in Section 3.3, we should analyze the time series at first. Through the white noise checking, the p-value of LB-statistics is 0.04, so the QoS series has serial dependency. Then, we can forecast the future values by the time series forecasting method. Through the stationary checking, the series can be regarded as a stationary time series. The ACF and PACF of this time series are shown in Figure 6, where the blue dashed lines denote the twice standard errors for autocorrelation coefficient and partial autocorrelation coefficient.

Though observing the characteristics of ACF and PACF, the following models can be constructed as the candidate models to the fitted time series, which are ARIMA(1,0,0), ARIMA(2,0,0), ARIMA(2,0,1) and ARIMA(3,0,1). After identifying candidate ARIMA models in the model estimation phase, the estimations of these models are listed in Table 2.

**Figure 7:**
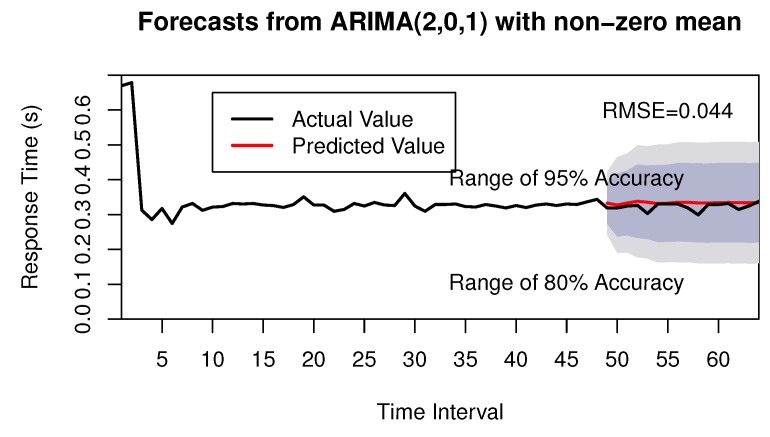
Forecasting of QoS Series. RMSE: root-mean-square error.

All of the identified models are estimated and their associated parameters are calculated in the model estimation phase. The best fitted model must be selected in the following phase. The procedure of model selection phase uses the minimum AIC value. From Table 2, we know the minimum value of AIC is −149.24, so the model of ARIMA(2,0,1) is selected as the best model. Through model checking, this model is found to be significant, so the ARIMA(2,0,1) can be selected to forecast the future values. Finally, the future values are obtained by the time series forecasting. The forecasting figure can be shown in Figure 7, where the red solid line presents the forecasting values by our approach, and the dashed area presents the range of different accuracy. Here we adopt the root-mean-square error (RMSE) to evaluate the accuracy of our forecasting approach, which can be calculated as follows.

(17) $$RMSE=\sqrt{\frac{\sum_{i=1}^{n}{\left(\hat{y_{i}}-y_{i}\right)}^{2}}{n}}$$

where *n* is the number of forecastings, and $y_{i}$ and $\hat{y_{i}}$ represent the real value and the forecasting value, respectively. After calculating the value of RMSE, we know the RMSE is 0.044; it is obvious that we can obtain the future QoS comparison values with high accuracy.

**Figure 8:**
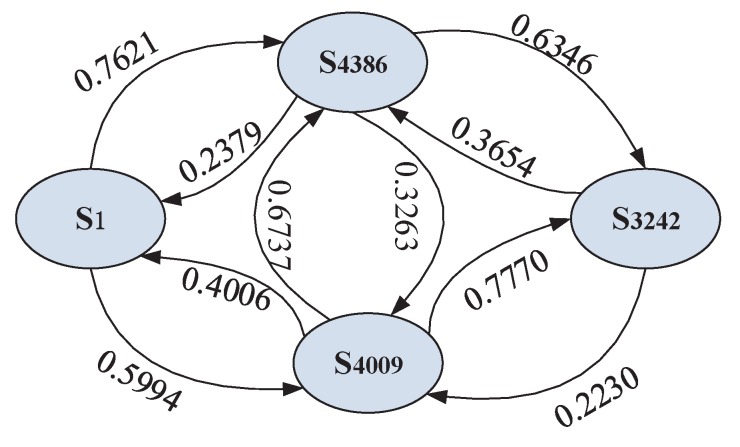
Case study of the markov chain.

Since the future comparison values are obtained by time series forecasting, we will construct the Markov chain based on the forecasting values. For instance, services with ID 4386 and 4009 are invoked by client 1 and 2, so the average of forecasting value 0.3263 is used to construct the Markov chain, which can be found in Figure 8. Finally, we can obtain the steady-state probabilities and sort them to derive the global ranking. The predicted global ranking is [S3242,S4386,S4009,S1], which is the same as the actual ranking.

### 5.2. Prototype System

In this paper, a time-aware service ranking prediction approach is introduced for obtaining the global service ranking, which can provide the accuracy service ranking of functionally similar services. This approach can recommend the optimal services to the users from a large number of available services for service selection and service composition. We believe this can help to build the high-quality IoT application systems. In order to investigate the benefits and practical application scenarios of our approach, a prototype system of IoT service publish platform is introduced.

**Figure 9:**
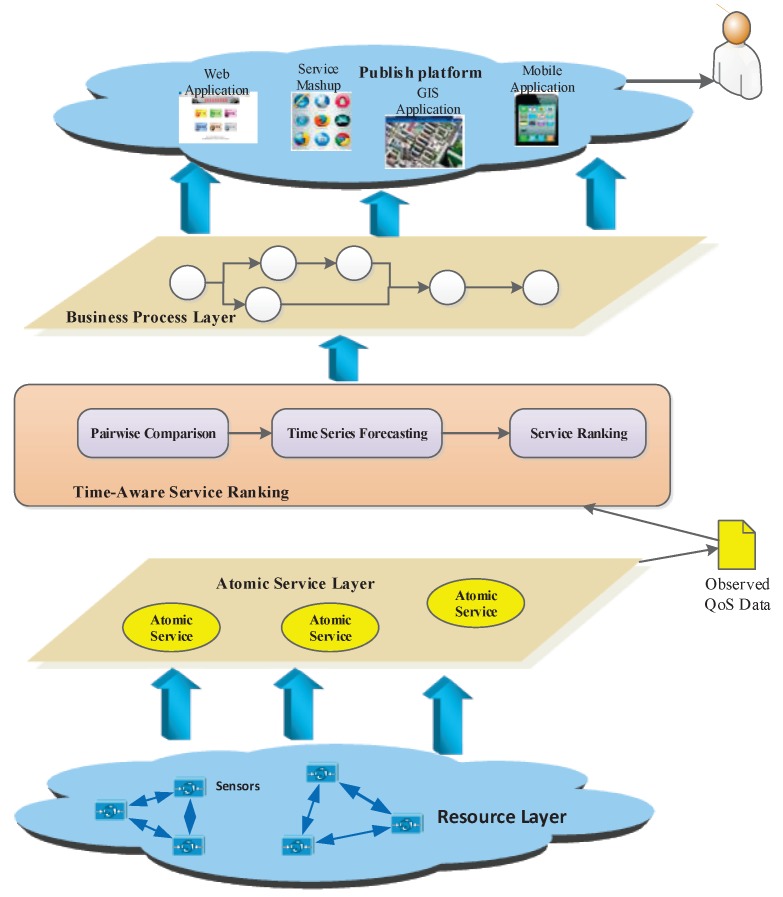
Framework of the prototype system.

Figure 9 shows an overall framework of an IoT service publishing platform, which can be divided into five layers. These five layers consist of the resource layer, atomic service layer, time-aware service ranking layer, business process layer, and public platform layer. The IoT service publishing platform is used to provide the public applications for users. In this system, the functionally similar services are selected to compose a series applications. The service ranking layer is the core component of this system. The detailed design of this prototype system is introduced as follows:

The resource layer is the lowest layer in this system. Devices, especially sensors, are used to collect the multi-type data, which are usually geographically dispersed in different locations. All of the data collected by the sensors provides the basic resource for atomic service. The atomic services are encapsulated in the atomic service layer, and most of them may be handled with large-volume data to complete a certain function. During the execution of these atomic services, the original QoS data are collected as the input of the service ranking layer. The service ranking layer provides the functionality for optimal, functionally similar candidate services, which analyze the original QoS data collected from different clients. Through a series manipulation of the QoS data described in above sections, the optimal services are selected and ranked, thus the atomic services are composed of the business process in the business process layer, and then the IoT applications are encapsulated by some conditions. Finally, different applications composed of some atomic services are published in the publishing platform, which can be used to build the high-quality systems.

In our previous work [18], we developed the District Heating Control and Information System (DHCIS) and the Coal Mine Comprehensive Monitoring and Early Warning System (CCMWS), which have been applied in reality. The DHCIS includes 200 monitoring boiler rooms and heat transfer stations in Beijing, which run in 120 heating districts. The CCMWS integrates the data and resources of the applications to ensure the safety of coal mine production, and can realize office automation for the computer client or mobile client. In these systems, there are some sub-systems, such as the billing system, monitoring system etc., all of which consist of numerous IoT applications. These applications are composed of some atomic services, which acquire the sensor data such as room temperature, water flow and gas concentration etc. The optimal services are selected throughout our approach and form the IoT applications, furthermore, these applications are published in the IoT service publishing platform. Finally, the high-quality systems were built accordingly.

As aforesaid, the IoT service publishing platform can provide different applications or systems for users, including the IoT-based web systems and mobile applications. In this prototype system, the service ranking module is the core component to provide the accuracy ranking of candidate services, which can help to build the high-quality IoT systems. We will present experimental results of our approach based on real-world datasets in Section 6.2.

## 6. Evaluation

### 6.1. Theoretical Analysis

In this paper, it is assumed there are *n* IoT services invoked by *m* clients. During the procedures of partial ranking, our approach is constructed based on a pairwise comparison model. Each two services need be ranked in our approach, where the future QoS values of each pair are forecasted by the time series approach, so the computation complexity is $O(|S_{j}{|}^{2})$, where the $S_{j}$ represents the number of ranked services from user *j*. Therefore, the overall computation complexity is $O(\max_{j}\left(\right|S_{j}{\left|\right)}^{2})$. Furthermore, the partial rankings need be collected, and the transition matrix is calculated, whose computation is $O(n^{2})$.

During the process of solving the DTMC, the iterative algorithm is adopted in our algorithm; the analysis of the computation complexity is difficult work. Here, we give a primitive analysis for it. To the our best knowledge, the iterative algorithm can converge on the stationary distribution. We assume the algorithm can converge in *k* steps, so our algorithm can be completed in $O(kn^{2})$ time. Furthermore, when *k* is small, some previous work such as MRRR can be completed in $O(n^{2})$ time [15]. In our approach, the final step is to sort the steady-state probabilities, which can be completed in O(nlogn).

In summary, the overall computation complexity of TSRPred is $O(n^{2})$, compared with the traditional approach evaluating all the services at every client, with a computation overhead of about O(mn). Our algorithms only pay the factor of $\frac{n}{m}$ during the process for obtaining the global services ranking. However, since every client only evaluates partial services, our algorithms can decrease the overhead of the evaluating clients.

### 6.2. Experimental Evaluation

#### 6.2.1. Datasets and Evaluation Metrics

To evaluate the efficiency and accuracy of TSRPred, large-scale real-world QoS datasets are required. In this paper, we adopt the WSDream dataset [16,17], which includes the response time and throughput of the services in reality. The datasets includes 4500 publicly available services that are geographically dispersed in 57 contries, and are invoked by 142 users over 16 h with a time interval lasting for 15 min. Thus the WSDream datasets are two sets of 142×4500×64 user-service-time matrices, including 30,287,610 observations for response time and throughput.

In the WSDream dataset, each of the 142 users keeps invocation of 4500 services over 64 time intervals; more than 30 million observations are collected. Through the analysis of these observations, we find that all the response times are within the range of 0∼20 s with the mean value of 3.165 s, while the throughputs lies in 0∼6726.833 kbps whose average value is 9.509 kbps. Here, we use two metrics to investigate the efficacy of our approach, which are the Kendall rank correlation coefficient and the probability density function (PDF) of ranking error.

*A. Kendall rank correlation coefficient:* The Kendall rank correlation coefficient (KRCC) is widely-used metric to evaluate the degree of similarity of two rankings [19]. It is assumed there are two rankings in the same services. The KRCC value can be calculated by:

(18) $$KRCC=\frac{C-D}{n(n-1)/2}$$

where *n* represents the number of services, and *C* and *D* represent the number of concordant pairs and the number of discordant pairs between two rankings, respectively.

*B. PDF of ranking error:* The probability density function (PDF) of ranking error in our experiment can describe the relative likelihood for the rank of each service. Here we adopt this function to show the uncertainty and variance of the experimental results.

#### 6.2.2. Experimental Results

In this paper, in order to show the efficacy and benefits of our approach, we conduct several experiments. In these experiments, only a part of the services is randomly selected from the real world dataset. We vary the proportions of selected services in each client from 5% to 50% with a step value of 5% to show our approach’s ability for handling different matrix densities. For example, 5% means that 95% services are randomly moved from a client and the remaining 5% services are ranked to predict the global ranking. Finally, the accuracy is evaluated using the two metrics introduced above.

**Figure 10:**
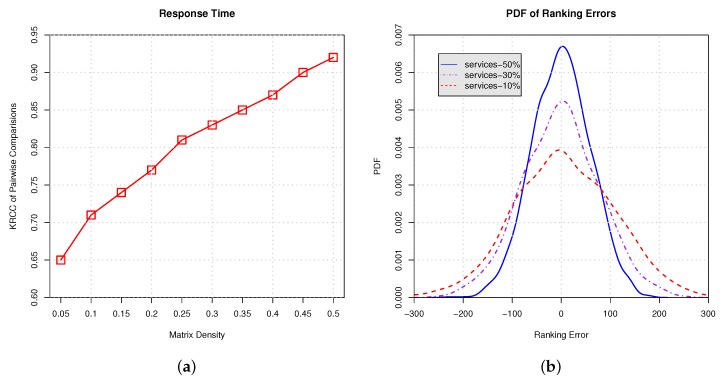
Ranking on response time with different proportions of services selection. (**a**) Kendall rank correlation coefficient (KRCC) of pairwise comparisons; (**b**) Probability density function (PDF) of ranking errors.

**Figure 11:**
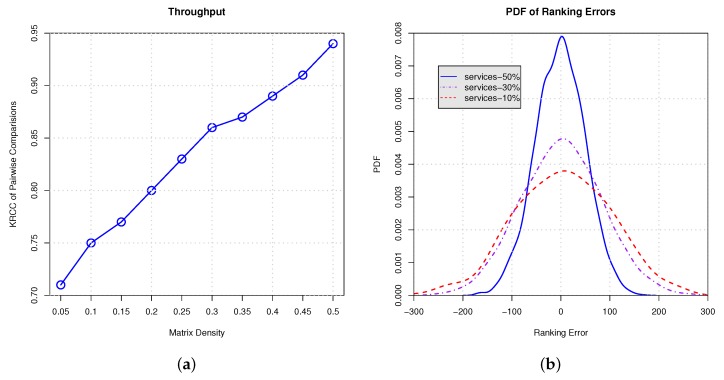
Ranking on throughput with different proportion of services selection. (**a**) KRCC of pairwise comparisons; (**b**) PDF of ranking errors.

In Figure 10 and Figure 11, we conduct empirical experiments on the ranking data using response time and throughput. Figure 10a and Figure 11a show the Kendall rank correlation coefficient (KRCC) of pairwise comparisons on response time and throughput, respectively. The results indicate that, with the increasing proportion of services selection, the KRCC goes up. Meanwhile, our approach can achieve good accuracy and binds the KRCC above 0.65 even when only 5% services are selected. Figure 10b and Figure 11b demonstrate the PDF of the ranking error on response time and throughput, which indicates that less services selection results in higher variance of the ranking prediction. All of the results show that our approach is able to derive the global ranking with acceptable errors.

## 7. Related Work

### 7.1. Service Ranking

Service ranking serves to sort a set of services with similar functionality according to some characteristics, which is an important and interesting issue in service-oriented computing, and is helpful in many aspects, e.g., service selection, service composition, and service recommendation, etc. With the scale of candidate services enlarged in the IoT environment, this issue is more significant.

For service ranking, most of the existing works ranked the services according to the QoS values. Several contributions have been proposed in service ranking, such as the Web services relevancy function (WsRF) [20] and simple additive weighting (SAW) [21], etc. Another type of service ranking is designed based on the similarity measurement, which has been proposed in some works. These methods include ranking the services using services network analysis [22,23] and component invocation frequency measurement [24], etc. The collaborative filtering (CF) approach is a notable exponent for ranking the services [25]. Some works have proposed efficient approaches based on collaborative filtering for service ranking in service recommendation. Zheng et al. [26] proposed a QoS-aware web service recommendation approach based on collaborative filtering. Tang et al. [27] proposed location-aware collaborative filtering for QoS-based service recommendation.

In summary, the study of service ranking focuses on the known QoS attributes and the relationship between different services. It is well known that obtaining the service ranking with acceptable accuracy under the inconsistent situation is a challenge. In this paper, we propose an approach to obtain the global service ranking.

### 7.2. QoS Prediction

As aforesaid, most of the service ranking approach is proposed based on the known QoS value or can be obtained from third organizations. However, it is difficult to do so since the QoS values may be unreliable or unknown. Therefore, many researchers have studied how to predict the QoS values. Collaborative filtering approaches are widely adopted in QoS prediction [28,29].

Some researchers have paid attention to handle with the temporal dynamics of QoS values. On one hand, the model-based CF approaches achieved time-aware QoS prediction by formalizing the problem as a user-service-time tensor factorization model [16,30]. On the other hand, the neighborhood-based approaches employ empirical weights to evaluate the joint impacts of historical QoS values over various time intervals for QoS prediction [31,32].

Besides collaborative filtering approaches, time series forecasting approaches have been successfully applied to modeling and forecasting QoS values [33,34]. They constructed different models to fit the known QoS values and then forecast the future changes. Godse et al. [35] proposed a time series model based on the ARIMA model to forecast service performance. Amin et al. [36] presented an improved method to fill the gaps of ARIMA models, which combines the ARIMA models and GARCH models. Li et al. [37] presented a comprehensive QoS prediction framework for composite services, which employs the ARMA model to predict the future QoS of individual service. Hu et al. [38] presented a novel personalized QoS prediction approach, which integrates the time series-based QoS forecasting for individual service. Ye et al. [39] proposed a novel approach to select and compose cloud services from a long-term and economic model-driven perspective, which uses QoS history value to predict the long-term QoS value.

In sight of these contributions, most mainly focused on QoS value prediction, ignoring the QoS ranking prediction. In this paper, we propose a time series based approach for service ranking prediction.

## 8. Conclusions

In this paper, we propose a time-aware service ranking prediction approach for obtaining the global service ranking from partial rankings. In this approach, the pairwise comparison model is constructed to describe the relationships between different IoT services, where the partial rankings are obtained by time series forecasting, and the comparisons of IoT services are formulated by random walks. Furthermore, the global ranking is obtained by sorting the steady-state probabilities of the Markov chain. Finally, the large-scale real world QoS dataset is adopted to validate the efficacy of our approach. We believe that our approach can help to build a high-quality IoT systems.

There are some avenues for our future work. Although our approach studies the temporal dynamics of QoS, the future values are forecasted based on the historical observations without any missing data. In reality, the historical observations may be missing in some cases, and thus how to obtain the service ranking with missing historical observations is an important problem in both academia and industry. Another goal of our future work is to design approaches to predict the service ranking considering the multi-dimensional QoS values. The relationships and tradeoffs between different QoS attributes should be carefully analyzed, and the ranking models as well as algorithms should be designed. 

## Figures and Tables

**Table 1:** Model Selection according to characteristics of ACF and PACF. ACF: autocorrelation function; PACF: partial autocorrelation function; AR: autoregressive; MA: moving average.

Models	ACF	PACF
AR(*p*)	Decays	Cuts off after lag p
MA(*q*)	Cuts off after lag q	Decays
ARMA(*p*,*q*)	Decays	Decays

**Table 2:** Estimations of candidate models. AIC: Akaike’s information criterion; ARIMA: autoregressive integrated moving average.

Model	Parameter	Estimation	Std. Error	AIC
ARIMA(1,0,0)	AR(1)	0.8137	0.1275	−135.97
ARIMA(2,0,0)	AR(1)	0.8545	0.1400	−139.65
	AR(2)	−0.7525	0.2251	
ARIMA(2,0,1)	AR(1)	0.2187	0.1394	−149.24
	AR(2)	−0.6506	0.2890	
	MA(1)	0.9349	0.0821	
ARIMA(3,0,1)	AR(1)	0.2469	0.1611	−147.36
	AR(2)	−0.6541	0.2946	
	AR(3)	0.1253	0.3684	
	MA(1)	0.9204	0.0896	
